# Evaluation of PiB visual interpretation with CSF Aβ and longitudinal SUVR in J-ADNI study

**DOI:** 10.1007/s12149-019-01420-2

**Published:** 2019-11-20

**Authors:** Yusuke Okada, Takashi Kato, Kaori Iwata, Yasuyuki Kimura, Akinori Nakamura, Hideyuki Hattori, Hiroshi Toyama, Kazunari Ishii, Kenji Ishii, Michio Senda, Kengo Ito, Takeshi Iwatsubo, Japanese Alzheimer’s Disease Neuroimaging Initiative

**Affiliations:** 1 Department of Radiology, Fujita Health University, 1-98 Dengakugakubo, Kutsukake-cho, Toyoake, 470-1192 Aichi Japan; 2 Department of Psychiatry, National Center for Geriatrics and Gerontology, 7-430 Morioka-cho, Obu, 474-8511 Aichi Japan; 3 Department of Clinical and Experimental Neuroimaging, National Center for Geriatrics and Gerontology, 7-430 Morioka-cho, Obu, 474-8511 Aichi Japan; 4 Department of Radiology, National Center for Geriatrics and Gerontology, 7-430 Morioka-cho, Obu, 474-8511 Aichi Japan; 5 Department of Radiology, Kindai University Faculty of Medicine, 377-2 Onohigashi, Osakasayama, 589-8511 Osaka Japan; 6 Diagnostic Neuroimaging Research, Tokyo Metropolitan Institute of Gerontology, 35-2 Sakae-cho, Itabashi-ku, Tokyo, 173-0015 Japan; 7 Division of Molecular Imaging, Kobe City Medical Center General Hospital, 2-1-1, Minatojimaminamimachi, Chuo-ku, Kobe, 650-0047 Hyogo Japan; 8 Department of Neuropathology, The University of Tokyo, 7-3-1, Hongo, Bunkyo-ku, Tokyo, 113-0033 Japan

**Keywords:** Alzheimer’s disease, Amyloid, ^11^C-PiB PET, Visual interpretation, Longitudinal

## Abstract

**Objective:**

The objectives of the present study were to investigate (1) whether trinary visual interpretation of amyloid positron emission tomography (PET) imaging (negative/equivocal/positive) reflects quantitative amyloid measurements and the time course of ^11^C-Pittsburgh compound B (PiB) amyloid accumulation, and (2) whether visually equivocal scans represent an early stage of the Alzheimer’s disease (AD) continuum in terms of an intermediate state of quantitative amyloid measurements and the changes in amyloid accumulation over time.

**Methods:**

From the National Bioscience Database Center Human Database of the Japanese Alzheimer’s Disease Neuroimaging Initiative, we selected 133 individuals for this study including 33 with Alzheimer’s disease dementia (ADD), 52 with late mild cognitive impairment (LMCI), and 48 cognitively normal (CN) subjects who underwent clinical assessment, PiB PET, and structural magnetic resonance imaging (MRI) with 2 or 3-years of follow-up. Sixty-eight of the 133 individuals underwent cerebrospinal fluid amyloid-β_1-42_ (CSF-Ab_42_) analysis at baseline. The standard uptake value ratio (SUVR) of PiB PET was calculated with a method using MRI at each visit. The cross-sectional values, longitudinal changes in SUVR, and baseline CSF-Ab_42_ were compared among groups, which were categorized based on trinary visual reads of amyloid PET (negative/equivocal/positive).

**Results:**

From the trinary visual interpretation of the PiB PET images, 55 subjects were negative, 8 were equivocal, and 70 were positive. Negative interpretation was most frequent in the CN group (70.8/10.4/18.8%: negative/equivocal/positive), and positive was most frequent in the LMCI group (34.6/1.9/63.5%) and in the ADD group (9.1/6.1/84.8%). The baseline SUVRs were 1.08 ± 0.06 in the negative group, 1.23 ± 0.15 in the equivocal group, and 1.86 ± 0.31 in the positive group (*F* = 174.9, *p* < 0.001). The baseline CSF-Ab_42_ level was 463 ± 112 pg/mL in the negative group, 383 ± 125 pg/mL in the equivocal group, and 264 ± 69 pg/mL in the positive group (*F* = 37, *p* < 0.001). Over the 3-year follow-up, annual changes in SUVR were − 0.00 ± 0.02 in the negative group, 0.02 ± 0.02 in the equivocal group, and 0.04 ± 0.07 in the positive group (*F* = 8.4, *p* < 0.001).

**Conclusions:**

Trinary visual interpretation (negative/equivocal/positive) of amyloid PET imaging reflects quantitative amyloid measurements evaluated with PET and the CSF amyloid test as well as the amyloid accumulation over time evaluated with PET over 3 years. Subjects in the early stage of the AD continuum could be identified with an equivocal scan, because they showed intermediate quantitative amyloid PET, CSF measurements, and the amyloid accumulation over time.

**Electronic supplementary material:**

The online version of this article (10.1007/s12149-019-01420-2) contains supplementary material, which is available to authorized users.

## Introduction

Alzheimer’s disease (AD) is the most common cause of dementia. Amyloid accumulation is predictive of a high probability of AD and is thought to begin before the appearance of clinical symptoms [1]. Amyloid positron emission tomography (PET) imaging using ^11^C-Pittsburgh compound B (PiB) has enabled visual and quantitative evaluation of cortical amyloid deposition in vivo [2, 3]. Time-course changes of amyloid deposition have been investigated using longitudinal semiquantitative values such as the standardized uptake value ratio (SUVR) of amyloid PET data. Groups with high accumulation and low accumulation of amyloid PET, which were divided according to cutoff values obtained by various methods, have demonstrated increasing and non-increasing deposition of amyloid, respectively [4–8]. Visual inspection may detect deposition of amyloid plaques earlier than semiquantitative methods [9]. However, there has been no research that stratified amyloid accumulation by visual interpretation in longitudinal analyses. In recent years, research interest has moved to detection of the earliest accumulation of amyloid and how it changes over time, as well as how it relates to neurodegeneration and the decline in cognitive function, since accurate understanding of the relationship between clinical status and the extent of progression of amyloid pathology is necessary to develop amyloid-targeted treatment plans.

In this study, the longitudinal changes of PiB PET in the Japanese Alzheimer’s Disease Neuroimaging Initiative (J-ADNI), a multicenter, prospective, observational study of Japanese subjects, were analyzed. In J-ADNI, visual interpretation of amyloid PET was performed based on trinary reading (negative, equivocal, or positive) according to strict reading criteria and a consensus process [10], which are different from the common visual interpretation of amyloid PET based on binary reading (negative or positive). With binary reading, the visual evaluation of PiB images can result in equivocal ratings [11]. With trinary reading, in which the judgement criterion of “equivocal” is defined, equivocal PET images may represent subtle accumulation in the earliest phase in the progression of amyloid accumulation that is likely to be overlooked by image interpretation using binary reading [12]. In J-ADNI, amyloid-β1-42 in cerebrospinal fluid samples (CSF-Ab_42_) was measured at baseline. Low CSF-Ab_42_ is considered the best biomarker that represents a pathologic state that is associated with amyloid plaque formation [1]. The objectives of the present study were to investigate (1) whether trinary visual interpretation of amyloid PET imaging (negative/equivocal/positive) reflects quantitative amyloid measurements and the time course of PiB amyloid accumulation, and (2) whether visually equivocal scans represent an early stage of the AD continuum [13] in terms of an intermediate state of quantitative amyloid measurements and time course of amyloid accumulation, as well as CSF-Ab_42_.

## Methods

Data used in preparation of this article were obtained from the J-ADNI database deposited in the National Bioscience Database Center (NBDC) Human Database, Japan (Research ID: hum0043.v1, 2016). The J-ADNI is a multicenter, longitudinal observational study of AD launched in 2007 as a public–private partnership, led by Principal Investigator Takeshi Iwatsubo, MD. In J-ADNI, individuals who were cognitively normal (CN), those with late mild cognitive impairment [14] (LMCI), and those with mild Alzheimer’s type dementia [15] (ADD) between the ages of 60 and 84 years were diagnosed and enrolled using inclusion and exclusion criteria harmonized with the Alzheimer’s Disease Neuroimaging Initiative [16]. Approval for the J-ADNI study protocol (UMIN000001374) was obtained from the local ethics committees or institutional review committees at the 38 participating clinical sites. A full description of the J-ADNI cohort has been reported elsewhere [17]. The latest data used in this study were downloaded on October 25, 2016. This study was approved by the Ethics Committee of the National Center for Geriatrics and Gerontology (no. 925-4). The authors have no competing interests to declare.

**Fig. 1:**
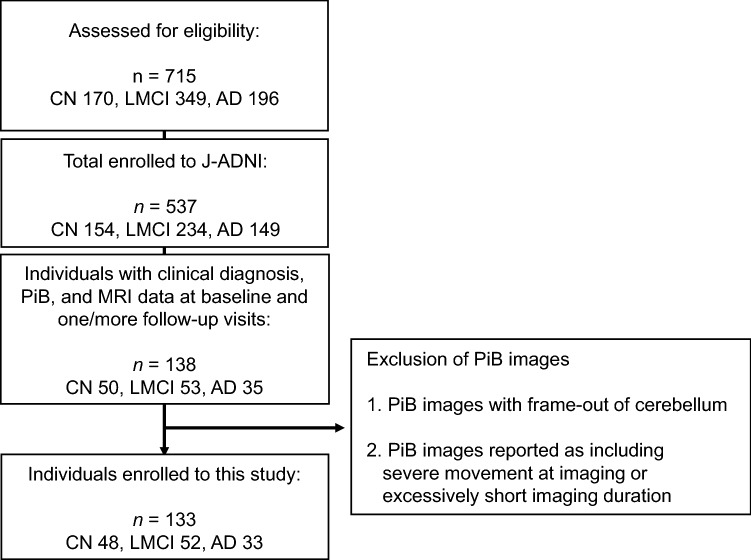
Diagram showing the number and flow of participants in this study

From the J-ADNI database, the individuals selected for this study included 33 with ADD, 52 with LMCI, and 48 who were CN. All study participants underwent neuropsychological assessments, PiB PET, and structural magnetic resonance imaging (MRI) (Fig. 1). The imaging examinations were performed, as a general rule, at baseline, 12 months, 24 months for all clinical categories, and 36 months for CN and LMCI between September 2008 and September 2014. The time interval between neuropsychological tests and PiB PET in each visit was 10.3 ± 13.2 days (mean ± standard deviation (SD)). The time interval between MRI and PiB PET was 14.5 ± 22.0 days (mean ± SD).

The basic characteristics of the 133 individuals stratified by clinical diagnoses are shown in Table 1. There were no significant differences in sex or ApoE ε2 or ε4 distributions among the groups. The ADD group was significantly older, had fewer years of school education, and scored worse on each neuropsychological examination than the LMCI and CN groups. The LMCI group was significantly older and scored worse on each neuropsychological examination than the CN group. In the present study, the number of individuals who underwent three or more PiB PET scans was 47 (97.9%) in the CN group, 49 (94.2%) in the LMCI group, and 28 (84.8%) in the ADD group. The duration of follow-up for PiB PET was 2.89 ± 0.37 years in CN, 2.69 ± 0.56 years in LMCI, and 1.79 ± 0.36 years in ADD.

**Table 1:** Demographic of the individuals

	CN (*n* = 48)	LMCI (*n* = 52)	ADD (*n* = 33)
Age, years	66.8 (4.5)	71.6 (5.1)^✝✝^	74.6 (6.8)^**☨^
Male sex, no. (%)	24 (50%)	24 (46.2%)	16 (48.5%)
Years of education	13.9 (2.3)	13.8 (2.9)	12.6 (3.3)^*^
ApoE ε2 + , no. (%)	4 (8.3%)	3 (5.8%)	1 (3%)
ApoE ε4 + , no. (%)	16 (33.3%)	24 (46.2%)	14 (42.4%)
CDR	0 (0)	0.5 (0)	0.6 (0.2)^**☨☨^
CDR-SOB	0 (0.1)	1.6 (1) ^✝✝^	3.5 (1.3)^**☨☨^
MMSE	29.5 (0.9)	26.6 (1.8) ^✝✝^	22.2 (1.6)^**☨☨^
ADAS	4.6 (2.7)	9.1 (4.3)^✝✝^	16.6 (4.6)^**☨☨^
CSF-Ab_42_ (pg/mL)^a^	425 (117)	324 (138)^✝^	272 (62)^**^
PiB visual interpretation			
Negative, No. (%)	34 (70.8%)	18 (34.6%)	3 (9.1%)
Equivocal, No. (%)	5 (10.4%)	1 (1.9%)	2 (6.1%)
Positive, No. (%)	9 (18.8%)	33 (63.5%)	28 (84.8%)
PiB SUVR	1.21 (0.28)	1.56 (0.43)^✝✝^	1.81 (0.41)^**☨^
ΔSUVR (/year)	0.01 (0.03)	0.03 (0.06)^✝✝^	0.02 (0.07)

Unless otherwise indicated, data are expressed as mean (SD). The items other than ΔSUVR shows data at baseline

*ApoE* apolipoprotein E, *CDR* Clinical Dementia Rating-Japanese, *CDR-SOB* Clinical Dementia Rating-Japanese sum of boxes, *MMSE* Mini-Mental State Examination, *ADAS* Alzheimer's Disease Assessment Scale-cognitive component-Japanese version, *ΔSUVR* annual change in SUVR

^*^^✝^Significantly different from their respective CN group

^☨^Significantly different from LMCI group

^**,^^✝✝,☨☨^*p* < 0.001

^*^^☨^*p* < 0.05, significance levels are shown without multiple comparisons

^a^The individuals with available CSF data were 25 (52.1%) in CN, 32 (61.5%) in LMCI, and 11 (33.3%) in ADD group

The ApoE genotype was determined by direct sequencing [18]. Clinical Dementia Rating (CDR) was performed for assessment of the clinical status in this study. CDR has been commonly used to assess the severity of dementia [19].

## Imaging

The 133 individuals underwent a PiB PET scan at baseline and additional scans every 12 months during the follow-up periods of 2 (ADD) or 3 years (CN and LMCI) at 11 sites that used a total of seven different PET scanner models by three vendors (2 GE Advance, 2 GE Discovery ST Elite, 20 Shimadzu Eminence G/X, 21 Shimadzu Eminence SOPHIA B/L, 40 Shimadzu HEADTOME V, 36 Siemens Biograph 16, and 12 Siemens ECAT ACCEL). Inter-site differences were minimized by standardizing the imaging protocol [20]. All PET images acquired at each PET site went through the J-ADNI PET quality control (QC) process [10, 20], in which head motion between frames was corrected before creating images of summed frames (sumframe images) of 50–70 min (300 s × 4 frames) after injection of PiB (555 ± 185 MBq). Correction of attenuation was processed by an additional 6-min transmission scan with segmentation for dedicated PET scanners or by a computed tomography (CT) scan for PET/CT scanners [10, 20]. Five PiB images (four images at baseline, one image at the follow-up period of 12 months) were excluded from this study because they included severe movement during imaging or an excessively short imaging duration (Fig. 1).

Structural MRI was performed with 1.5-T MRI scanners using a three-dimensional sagittal magnetization-prepared rapid gradient-echo imaging (MPRAGE) sequence according to a standardized protocol [18] at each PiB follow-up period. For the 133 individuals, a total of 5 MRI scanner models by three vendors were used (34 GE SIGNA EXCITE, 17 GE SIGNA HDxt, 11 Philips Intera, 30 Siemens Avanto, 41 Siemens Symphony).

## Imaging procedures

### Visual interpretation

The PiB PET images generated through the QC process above were independently interpreted visually by three expert raters blinded to the clinical category. Information about age, sex, and T1-weighted MRI images was provided to the raters. In the visual interpretation, the raters evaluated the regional PiB uptake for each of four cortical areas on each side (frontal lobe, lateral temporal lobe, lateral parietal lobe, and precuneus/posterior cingulate gyrus) as positive, equivocal, or negative regional uptake, the definitions of which were as follows: positive, uptake is clearly higher than in cerebral white matter that covers more than one gyrus of the cerebral cortical area; equivocal, uptake is slightly higher than or similar to that in cerebral white matter that covers more than one gyrus of the cerebral cortical area, i.e., radioactivity extending beyond white matter to the cortical surface, or a high uptake spot limited to one gyrus; and negative, uptake is lower than in cerebral white matter in any region of the cerebral cortex. Of the total scans in J-ADNI, 91.3% of the visual assessments were matched among the three raters (Cohen kappa = 0.88). After independent interpretations, consensus reading was performed to determine the unified visual interpretation for each PET image by the three raters and two other experienced discussants [10]. The unified visual interpretation stored in the J-ADNI was used for this study.

### Quantitative evaluation

MPRAGE MR images that were interpolated were converted into images of a cubic voxel (1.2 × 1.2 × 1.2 mm^3^) using the Voxel-based Specific Regional analysis system for Alzheimer’s Disease (VSRAD) [21]. The sumframe images of PiB were coregistered to the individual MR images using Pmod 3.4 (https://www.pmod.com/web/) with a combination of methods (dissimilarity function: normalized mutual information; interpolation of pixel values: trilinear; resampling density: 5.2 mm; minimization method: Powell’s method [22]). The cubic voxel MRI images were segmented into grey matter images using the Statistical Parametric Mapping software package (SPM8) (https://www.fil.ion.ucl.ac.uk/spm/software/spm8/). The coregistered PiB images and grey matter-segmented MR images were spatially normalized in stereotactic space of the Montreal Neurological Institute (MNI) 152 template [23] using Diffeomorphic Anatomical Registration using Exponentiated Lie Algebra (DARTEL) [24], with parameters obtained from the individual MR images. Grey matter-mask images were made with binarization from the spatially normalized grey matter-segmented MR images using an in-house program. The normalized PiB PET images were masked with the grey matter-mask MR images to exclude the white matter and regions outside the brain. The region of interest (ROI) values were obtained from the PiB images of the grey matter using the Automated Anatomical Labeling Atlas [25]. All ROI values were transformed into SUVRs by dividing them by the average ROI value in the cerebellar cortex as reference, because 6-CN-PiB binding to diffuse amyloid plaques in the cerebellum cortex was not detectable in a pathological study [2]. The mean cortical SUVR was obtained by averaging the ROI values of the frontal, parietal, and temporal ROIs [26, 27].

### CSF biomarkers

A total of 68 (11 ADD, 32 LMCI, 25 CN) individuals in this study underwent a lumbar puncture at baseline. CSF-Ab_42_ in the CSF samples was assayed using the multiplex xMAP Luminex platform (Luminex Corp, Austin, TX) with Innogenetics (INNO-BIA AlzBio3; Ghent, Belgium) immunoassay kit-based reagent as validated previously [28] at the J-ADNI biomarker core at Niigata University [17].

### Statistical analyses

The subject’s characteristics at baseline were summarized by frequency and percentage for categorical variables and by means and SDs for continuous variables. Group comparisons were made by Fisher’s exact test for categorical variables and the *t *test for continuous variables. Single regression analyses of SUVRs at available time points for each participant were performed to calculate the annual increase in SUVRs. For reference, the upper confidence limit of the 97.5 percentile for the baseline SUVR in the amyloid-negative CN group was calculated. The mean/SD of the SUVR at baseline and the annual change in the SUVR were calculated for each group corresponding to each visual judgment and clinical category. Statistical analysis of baseline SUVR, annual change in SUVR, and baseline CSF-Ab_42_ levels were compared by one-way analysis of variance, followed by post-hoc comparisons using the Bonferroni correction. Statistical analyses were performed using SPSS version 25.

## Results

With trinary visual interpretation of the PiB PET images, 55 subjects were negative, eight were equivocal, and 70 were positive. In the CN group, negative was most frequent (34/48, 70.8%), whereas five participants were equivocal (10.4%), and nine were positive (18.8%). In the LMCI group, positive was most frequent (33/52, 63.5%), whereas 18 participants were negative (34.6%), and one was equivocal (1.9%). In the ADD group, positive was most frequent (28/33, 84.8%), whereas three participants were negative (9.1%), and two were equivocal (6.1%). The PiB SUVR was significantly different among the groups (CN 1.21 ± 0.28; LCMI 1.56 ± 0.43; ADD 1.81 ± 0.28). The SUVR change per year was significantly higher in the LCMI group than in the CN group, but the annual change of SUVR changes in the ADD group did not differ from those in the LMCI and CN groups. CSF-Ab_42_ was significantly higher in the CN group than in the LMCI and ADD groups, but no significant difference between the LMCI and ADD group was found (Table 1). Trinary visual interpretation in the CN group did not define the course of clinical symptoms. Two of 34 subjects in the negative group, zero of five subjects in the equivocal group, and one of nine subjects in the positive group showed worsening of clinical symptoms from CDR 0 to 0.5.

**Fig. 2:**
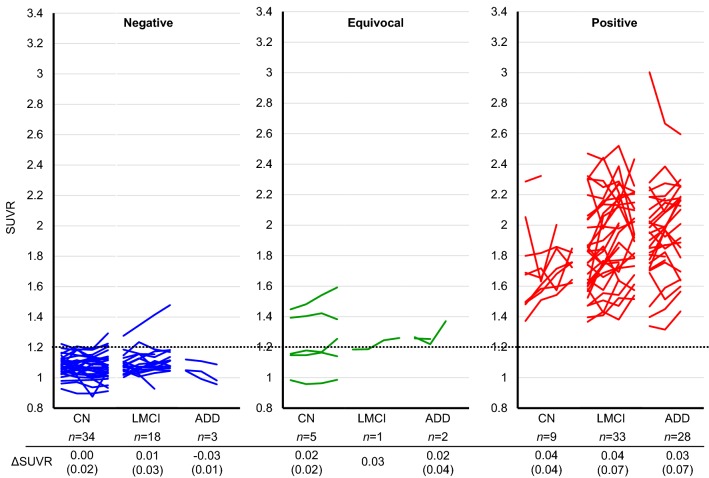
Individual longitudinal changes in SUVR during the 3-year follow-up. Scatterplots show the SUVR at baseline and in the 3-year follow-up. The dotted line shows the SUVR threshold of 1.20 derived from the upper confidence limit of the 97.5 percentile for the baseline SUVR in the amyloid-negative CN group. *Negative* PiB visually amyloid-negative, *Equivocal* PiB visually equivocal, *Positive* PiB visually amyloid-positive, *ΔSUVR* the annual change in the SUVR

Figure 2 shows the time courses of SUVRs grouped by visual assessment and by clinical category. In the amyloid-negative CN group, the upper confidence limit of the 97.5 percentile for the baseline SUVR was 1.20. Most SUVRs in the amyloid-negative group (LMCI, ADD) remained below this level. In the amyloid-equivocal group, no evident differences in the longitudinal trends of SUVR were observed among the CN, LMCI, and ADD groups. In the amyloid-positive group, all individual values remained above SUVR 1.3, indicating a clear difference in the distribution of the SUVR between the amyloid-positive and amyloid-negative groups. In the visually amyloid-positive group, the annual changes in the SUVR were 0.04 ± 0.04 in the CN group, 0.04 ± 0.07 in the LMCI group, and 0.03 ± 0.07 in the ADD group. The SUVRs in the amyloid-positive CN group tended to increase over time from a lower baseline SUVR compared with the LMCI and ADD groups. Conversely, no evident differences in the longitudinal trends of SUVR were observed among the LMCI and ADD groups. In the visually amyloid-positive group, 19 individuals showed a decreasing SUVR (CN *n* = 1, LMCI *n* = 8, ADD *n* = 10). The baseline SUVR in these individuals was significantly higher than the baseline SUVR of all visually amyloid-positive individuals (2.10 ± 0.33 vs 1.86 ± 0.31, *p* = 0.003).

**Fig. 3:**
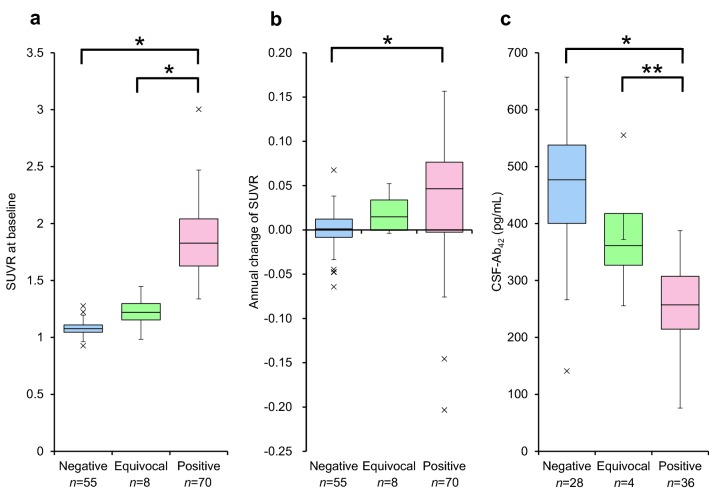
Box-plots of longitudinal changes in SUVR and the baseline CSF-Ab_42_ level in the visual interpretation groups. **a** Box-plots show the baseline SUVR. **b** Box-plots show the annual changes in SUVR. **c** Box-plots show the baseline CSF-Ab_42_ level in individuals with available CSF data. The SUVR at baseline in the visually amyloid-positive group was significantly higher than that in the amyloid-negative group and amyloid-equivocal group (*p* < 0.001). The annual change in the SUVR in the visually amyloid-positive group was significantly higher than that in the amyloid-negative group (*p* < 0.001). The CSF-Ab_42_ level at baseline in the visually amyloid-positive group was significantly lower than that in the amyloid-negative group (*p* < 0.001) and the amyloid-equivocal group (*p* < 0.05). *p < 0.001, **p < 0.05, *Negative* PiB visually amyloid-negative, *Equivocal* PiB visually equivocal, *Positive* PiB visually amyloid-positive

Baseline SUVR, annual changes in the SUVR, and the baseline CSF-Ab_42_ level in the visual interpretation groups are presented as mean/SDs (Fig. 3). Comparison of groups stratified by visual assessment alone showed that the baseline SUVRs were 1.08 ± 0.06 in the negative group, 1.23 ± 0.15 in the equivocal group, and 1.86 ± 0.31 in the positive group (*F* = 174.9, *p* < 0.001). The annual change in SUVR was − 0.00 ± 0.02 in the negative group, 0.02 ± 0.02 in the equivocal group, and 0.04 ± 0.07 in the positive group (*F* = 8.4, *p* < 0.001). The baseline CSF-Ab_42_ level was 463 ± 112 pg/mL in the negative group, 383 ± 125 pg/mL in the equivocal group, and 264 ± 69 pg/mL in the positive group (*F* = 37, *p* < 0.001).. The differences in baseline SUVR, the annual change in SUVR, and the CSF-Ab_42_ level between the negative and positive groups were significant (*p* < 0.001). The mean baseline SUVR, annual change in SUVR, and CSF-Ab_42_ level in the amyloid-equivocal group were between those in the amyloid-negative and -positive groups. The differences in baseline SUVR and the CSF-Ab_42_ level between the equivocal and positive groups were significant (*p* < 0.001, *p* < 0.05), although the difference in the annual change in SUVR was not significant (*p* = 1.00). The differences in all parameters were not significant between the amyloid-equivocal and amyloid-negative group (*p* = 0.29 for baseline SUVR, *p* = 0.87 for annual change of SUVR, and *p* = 0.33 for CSF-Ab_42_).

**Fig. 4:**
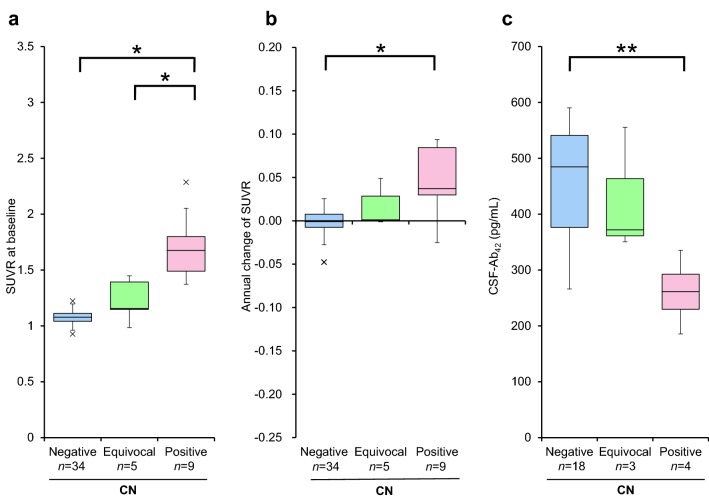
Box-plots of longitudinal changes in SUVR and the baseline CSF-Ab_42_ level in CN individuals. **a** Box-plots show the baseline SUVR. **b** Box-plots show the annual changes in SUVR. **c** Box-plots show the baseline CSF-Ab_42_ of CN individuals with available CSF data. In CN individuals, the SUVR at baseline in the amyloid-positive group was significantly higher than that in the amyloid-negative group and amyloid-equivocal group (*p* < 0.001). The annual change in the SUVR in the amyloid-positive group was significantly higher than that in the amyloid-negative group (*p* < 0.001). The CSF-Ab_42_ level at baseline in the amyloid-positive group was significantly lower than that in the amyloid-negative group (*p* = 0.003). *p < 0.001, **p = 0.003, *Negative* PiB visually amyloid-negative, *Equivocal* PiB visually equivocal, *Positive* PiB visually amyloid-positive

Additionally, we performed an analysis with the CN group to see the effect of trinary visual interpretation in subjects in the very early stage of the AD continuum. The differences in baseline SUVR, annual changes in the SUVR, and baseline CSF-Ab_42_ levels in CN individuals were evaluated in terms of visual interpretation (Fig. 4). In CN individuals, the baseline SUVRs were 1.08 ± 0.06 in the negative group, 1.23 ± 0.19 in the equivocal group, and 1.70 ± 0.30 in the positive group (*F* = 63.4, *p* < 0.001). The annual change in SUVR was − 0.00 ± 0.02 in the negative group, 0.02 ± 0.02 in the equivocal group, and 0.04 ± 0.04 in the positive group (*F* = 13.0, *p* < 0.001). The baseline CSF-Ab_42_ level was 461 ± 97 pg/mL in the negative group, 426 ± 113 pg/mL in the equivocal group, and 261 ± 63 pg/mL in the positive group (*F* = 7.3, *p* = 0.004). The differences in baseline SUVR, the annual change in SUVR, and the CSF-Ab_42_ level between the negative and positive groups were significant (*p* < 0.001 for baseline SUVR, *p* < 0.001 for annual change of SUVR, and *p* = 0.003 for CSF-Ab_42_). The differences in baseline SUVR between the equivocal and positive groups were significant (*p *< 0.001), although the difference in the annual change in SUVR and the CSF-Ab_42_ level was not significant (*p* = 0.13, *p* = 0.10). No significant differences were observed in these parameters between the amyloid-equivocal group and the amyloid-negative group (*p* = 0.13 for baseline SUVR, *p* = 0.41 for annual change of SUVR, and *p* = 1.00 for CSF-Ab_42_). However, these parameters in the amyloid-equivocal group had a tendency to distribute between the amyloid-negative and amyloid-positive groups.

## Discussion

In recent years, the detection of the earliest accumulation of amyloid has received great emphasis, because it is necessary to accurately determine the progression of amyloid pathology [29], and develop amyloid-targeted therapeutic protocols [30]. When determining the cutoff value of the mean cortical SUVR for positive and negative judgement, performing a receiver operating characteristic using the clinical diagnosis as standard of truth is common [31, 32]. However, cutoff levels determined in this manner do not detect the earliest accumulation of amyloid, because the SUVR is the average of cortical uptake and may not reflect localized and/or weak but abnormal uptake [9]. Attempts have been made to determine the cortical mean accumulation that corresponds to a subtler accumulation [29, 33, 34]. Visual reading may be more sensitive to focal and asymmetric increases in PiB than semiquantitative method, although this sensitivity could produce false positives [9]. Visual reading may interpret such cases as equivocal. In many cases, definitive visually-positive or -negative PET images are presented, but a few cases present with images that are neither positive nor negative. In the J-ADNI study, such PET images were defined as equivocal, and visual readings of PiB PET were performed using a trinary reading method for evaluating negative, equivocal, and positive scans [10]. Images visually interpreted as equivocal in the J-ADNI may be judged negative if they are interpreted visually by applying the binary reading criteria in F-18 labeled amyloid tracers. In this study, we used the longitudinal data of CSF-Ab_42_ and PiB PET to verify whether this equivocal determination can detect a subtle initial accumulation of amyloid.

When the three clinical groups (ADD, LMCI, and CN) were combined, the baseline SUVR, annual change in the SUVR, and CSF-Ab_42_ levels were statistically compared among the visually amyloid-negative, visually equivocal, and the visually amyloid-positive groups (Fig. 3). A one-way analysis of variance showed a significant difference in the mean values of the three groups. The amyloid-equivocal group had intermediate parameter values that were between the negative and positive groups. Statistical analysis of the CN group alone also showed the same results (Fig. 4). The visually amyloid-equivocal group was identified as having an intermediate status of amyloid pathology in terms of the SUVR, its annual change, and the biological indicator, CSF-Ab_42_. These results suggest that individuals with visually equivocal scans are at higher risk of progression of amyloid accumulation compared to those with visually amyloid-negative scans.

In individuals evaluated as visually amyloid-negative, their SUVRs remained within the 97.5 percentile of the baseline SUVR over time. This finding was observed regardless of the clinical categories of CN, LMCI, and ADD (Fig. 2). Barring a few exceptions, individuals with an amyloid-negative image showed no trend towards a gradual increase in the SUVR. In the amyloid-negative group, very few individuals showed an upward trend in the SUVR. Similar cases have been found in other longitudinal cohorts [4, 35], suggesting that they may represent the very early stage when amyloid accumulation has turned upward. On the other hand, individuals with a visually amyloid-positive image showed a time course with an SUVR of 1.3 or higher. The SUVR in the visually amyloid-positive group showed a gradual increase with a few exceptions. This was consistent with previous studies that reported [4–7, 36, 37] that when a cut-off value of SUVR or distribution volume ratio dividing low and high accumulation was used, the high accumulation group showed a gradual increasing tendency above the cut-off value, and the low accumulation group did not show an increasing tendency under the cut-off value. These findings suggest that the risk of progression of amyloid accumulation is low in the visually-negative group, whereas the visually-positive group is at an increased risk as well as SUVRs.

In the present study, some individuals with relatively high SUVR in the visually amyloid-positive group showed decreased SUVR during follow-up. Similar reports have been made in the past, but the reasons are unclear [4, 36, 38]. In general, amyloid deposition may demonstrate a sigmoidal increase and reach a plateau around maximum accumulation [4, 38]. This finding means that the rate of amyloid deposition slows after reaching the peak speed in the progression of amyloid pathology. PiB accumulation may decrease for the following reasons: (1) slowing of the accumulation rate (2) partial volume effect due to progressive brain atrophy (3) statistical errors, and (4) unknown pathological changes that reduce amyloid plaques. The contribution of errors in calculating SUVR cannot be excluded. However, considering that the test–retest variability of SUVR is about 3–7%, and the decrease was observed at consecutive time intervals [39], the cause of the continuous decrease cannot be attributed to only the reproducibility of SUVR.

SUVRs can vary depending on the method of calculation, such as the ROI setting, and they are problematic when comparisons are made between studies. The SUVR of 1.2–1.3, which was considered a cutoff corresponding to visual interpretation of negative/positive in the present study (Fig. 3), was lower than the value of 1.5 reported by Yamane et al. [10]. SUVRs were calculated using an in-house method [26, 27], because the SUVRs used by Yamane et al. are not available in the NBDC database. The disagreement was thought to be caused by the difference in the setting of ROIs, such as using the Automated Anatomical Labeling [25] atlas and excluding areas other than cortices segmented with MRI.

ApoE ε4 is considered to be an important risk factor for amyloid deposition [40]. As shown in the Online resource most individuals in the amyloid-negative group did not carry ApoE ε4. In the amyloid-negative CN group, the annual change in the SUVR was significantly higher in ApoE ε4 carriers [0.01 ± 0.02 (mean ± SD)] than in non-carriers (− 0.01 ± 0.02) (*p* = 0.02). ApoE ε4 carriers were deemed to represent an increasing SUVR, even though ApoE ε4 carriers were visually amyloid-negative. On the other hand, presenting firm findings in other groups was difficult, because the sample size was insufficient for statistical investigation of the effect of ApoE ε4. For reference, a statistical summary of ε4 carriers and non-carriers in each group is provided (Online resource).

PiB PET visual interpretation is inevitably affected by each rater’s experience and potential personal bias. In the overall individuals in the J-ADNI study, agreement in the trinary visual interpretation (negative/equivocal/positive) was 91.3% among the three raters (Cohen kappa = 0.88) [10]. The interpretation of equivocal cases was not indistinct from the others, as the agreement rate and Cohen kappa of binary visual interpretation (negative/positive) did not differ from the trinary interpretation (92.3% and 0.89%, respectively).

Whether the results obtained with PiB PET can be applied to F-18-labeled amyloid PET is unclear, because PiB has a different contrast from F-18 amyloid tracers.

Our study has several limitations. First, the sample size was relatively small, and the follow-up duration was relatively short for observing changes in clinical status, especially for tracking CN individuals, although we implemented all available data from the J-ADNI study. Further validation in other cohort studies is needed.

Second, the results were potentially influenced by several methodological factors. We did not apply partial volume correction (PVC) in this study. This was because Villemagne et al., in their longitudinal PiB-PET study [4], employed data without PVC for the main results, as they found that PVC induced considerable noise and increased variances in their data. However, SUVRs in the current study could be affected by brain atrophy. Also, computation of SUVRs can introduce errors, and the methodology itself as well as within-subject variability in his/her general condition such as brain circulation can introduce errors. Engler et al. studied the test–retest variability of PiB-PET SUVRs in four subjects within 20 days, and found variances of 3–7% [39]. Thus, the large variances found in the current study need to be interpreted while considering the influence of these factors.

## Conclusion

Trinary visual interpretation (negative/equivocal/positive) of amyloid PET imaging reflects quantitative amyloid measurements evaluated by PET and the CSF amyloid test as well as the amyloid accumulation over time evaluated by PET over the 3 years. Subjects in the early stage of the AD continuum could be identified when they showed an equivocal scan, because they showed intermediate quantitative amyloid PET and CSF measurements as well as the amyloid accumulation over time.

## Electronic supplementary material

Below is the link to the electronic supplementary material.

Supplementary material 1 (DOCX 133 kb)
