# Supplementary material for: Evaluation of PiB visual interpretation with CSF Aβ and longitudinal SUVR in J-ADNI study
Source: Ann Nucl Med. 2019 Nov 20;34(2):108–18. doi: 10.1007/s12149-019-01420-2 (PMC7026272; doi:10.1007/s12149-019-01420-2)
Supplement: Supplementary file 1 — Supplementary material 1 (DOCX 133 kb) [file 12149_2019_1420_MOESM1_ESM.pdf]

Online Resource The detailed characteristics of ApoE ε4 carriers and non-carriers by clinical and PiB visual group[a]

	CN		LMCI		ADD	
	ε4 non-carrier	ε4 carrier	ε4 non-carrier	ε4 carrier	ε4 non-carrier	ε4 carrier
**Negative** (n=55)						
n =	26	8	18		3	
Age, years	66 (4.7)	65.6 (4.5)	72.2 (5.5)		77.8 (2.1)	
Gender, Male (%)	15 (57.7%)	4 (50%)	11 (61.1%)		2 (66.7%)	
Years of Education	14.6 (2.6)	13.6 (2)	14.7 (2.3)		11.7 (7.4)	
CDR-SOB	0.1 (0.2)	0 (0)	1.6 (1.1)		3.2 (1.2)	
MMSE	29.7 (0.6)	29.3 (0.9)	26.8 (1.9)		22.3 (1.5)	
ADAS	4.2 (2.5)	3.8 (2)	6.6 (2.6)		12 (4.4)	
PiB SUVR	1.08 (0.07)	1.08 (0.05)	1.09 (0.06)		1.07 (0.04)	
ΔSUVR (/year)	-0.01 (0.02)	0.01 (0.02)	0.01 (0.03)		-0.03 (0.01)	
**Equivocal** (n=8)						
n =	4	1		1	2	
Age, years	70.8 (3.7)	71.7 (0)		62.4	79.3 (7.3)	
Gender, Male (%)	2 (50%)	1 (100%)		0 (0%)	0 (0%)	
Years of Education	12.3 (0.5)	12 (0)		12	10.5 (2.1)	
CDR-SOB	0 (0)	0 (0)		1	5 (0)	
MMSE	29 (1.4)	29 (0)		28	21.5 (0.7)	
ADAS	4.5 (3.2)	7.3 (0)		10	20.9 (3)	
PiB SUVR	1.18 (0.19)	1.39 (0)		1.18	1.26 (0.01)	
ΔSUVR (/year)	0.02 (0.02)	0 (0)		0.03	0.02 (0.04)	
**Positive** (n=70)						
n =	2	7	10	23	14	14
Age, years	70.9 (3.8)	66.8 (3)	69.7 (5.8)	71.1 (4.6)	75.1 (8.3)	72.7 (5.4)
Gender, Male (%)	0 (0%)	2 (28.6%)	3 (30%)	11 (47.8%)	6 (42.9%)	8 (57.1%)
Years of Education	12 (0)	13.6 (1.4)	13.6 (1.8)	13.9 (3.3)	13.3 (2.7)	12.4 (3)
CDR-SOB	0 (0)	0 (0)	0.5 (0.6)	1.6 (0.8)	3.4 (1.6)	3.5 (1)
MMSE	30 (0)	29.1 (1.5)	28.1 (2.1)	26.8 (1.7)	22.1 (1.9)	22.4 (1.5)
ADAS	6.8 (4.9)	5.8 (3.1)	6.3 (3.9)	10.5 (3.9)	16.1 (4.4)	17.5 (4.5)
PiB SUVR	1.43 (0.09)	1.78 (0.3)	1.79 (0.33)	1.85 (0.28)	1.83 (0.29)	2.03 (0.33)
ΔSUVR (/year)	0.06 (0.03)	0.04 (0.04)	0.04 (0.05)	0.04 (0.07)	0.04 (0.05)	0.02 (0.09)

a; Individuals with ApoE ε4 genotype were treated as ApoE ε4 carriers and without this genotype they were treated as non-carriers. The items other than ΔSUVR shows data at baseline. Unless otherwise indicated, data are expressed as mean (SD). Abbreviations: ApoE, apolipoprotein E; CDR-SOB, Clinical Dementia Rating-Japanese sum of boxes; MMSE, Mini-Mental State Examination; ADAS, Alzheimer's Disease Assessment Scale-cognitive component-Japanese version; ΔSUVR, annual change of SUVR
